# Evolution of blood pressure management in acute intracerebral hemorrhage

**DOI:** 10.12688/f1000research.11687.1

**Published:** 2017-11-21

**Authors:** Stacy Chu, Lauren Sansing

**Affiliations:** 1 Department of Neurology, Yale School of Medicine, New Haven, CT, USA

**Keywords:** intracerebral hemorrhage, hypertension, stroke

## Abstract

Intracerebral hemorrhage (ICH) remains a prevalent and severe cause of death and disability worldwide. Control of the hypertensive response in acute ICH has been a mainstay of ICH management, yet the optimal approaches and the yield of recommended strategies have been difficult to establish despite a large body of literature. Over the years, theoretical and observed risks and benefits of intensive blood pressure reduction in ICH have been studied in the form of animal models, radiographic studies, and two recent large, randomized patient trials. In this article, we review the historical and developing data and discuss remaining questions surrounding blood pressure management in acute ICH.

## Introduction

Elevated blood pressure (BP) is prevalent in an estimated 60–84% ^1, 2^ of patients presenting with acute stroke ^2^. The pathophysiologic mechanisms underlying the acute hypertensive response observed in stroke are not clearly understood, although proposed theories have included autoregulation to improve cerebral perfusion, Cushing reflex in patients with elevated intracranial pressure (ICP), damage to brain areas regulating BP ^3^, and a sympathetic response to discomfort or illness ^4^. It is also reasonable to attribute at least a proportion of hypertension in patients with acute stroke to chronic hypertension, although this explanation would be inconsistent with the observed patterns of spontaneous reduction in BPs over the several days following stroke ^1^. Of stroke subtypes, there is a higher prevalence of severe hypertension among patients with acute intracerebral hemorrhage (ICH) ^2^. Interestingly, the Oxford Vascular Study found that the mean first systolic blood pressure (SBP) after ICH onset was much higher than the most recent pre-ICH reading, and this difference was greater than that seen in patients with acute ischemic stroke despite similar rates of premorbid hypertension (ICH mean 43.5 mm Hg increase versus ischemic stroke mean 17.9 mm Hg increase, *P* <0.0001) ^5^. Additionally, BPs decreased more rapidly in the first 24 hours after ICH than after ischemic stroke (41.4 versus 13.6 mm Hg, *P* = 0.0007), even after excluding patients who received antihypertensives and who died prior to 24 hours after onset. This suggests that the hypertensive response to ICH is different from other types of brain injury, perhaps due to the mass effect of the hemorrhage or responses to blood vessel rupture or blood products.

The clinical significance of hypertension in acute stroke and the optimal strategies for managing it have been topics of extensive study for many decades. Elevated admission BP has been associated with increased mortality and disability among certain groups of stroke patients ^6, 7^ and in particular among patients with ICH ^8^. A relationship between uncontrolled BP and hematoma volume expansion has been reported in several studies ^9– 11^; however, others have failed to find a clear association ^12, 13^. These conflicting data cast doubt on whether it is a simple linear relationship between pressure and hemorrhage growth. There are more consistent data on the association between hematoma growth and poor outcome ^13, 14^, so that any potential decrease in the risk of expansion attributable to aggressive BP control has therapeutic interest. The management of elevated BP in acute ICH confronts two conflicting pathophysiologic processes. First, BP reduction lowers hydrostatic pressure and therefore may attenuate hematoma expansion and theoretically perihematoma edema as well. Second, there is the potential risk of worsening ischemia in the perihematomal region or precipitating renal injury by aggressively lowering perfusion pressures. In the following paragraphs, we describe the main concerns that have surrounded BP management in acute ICH in recent years, and we summarize the data that have influenced various perspectives on this controversial topic.

## Summary of data and recent guidelines

### Safety

Recently, a number of clinical trials have demonstrated the relative safety of BP reduction in acute ICH. However, evidence of therapeutic benefit has remained elusive or variable, and optimal strategies to lower BP are poorly defined. Prior to large clinical trials, caution against aggressive BP treatment came in the form of retrospective data suggesting that rapid BP reduction, as reflected by steeper slopes of the change in mean arterial pressure (MAP) over time, was associated with increased mortality ^15^. Magnetic resonance imaging (MRI) diffusion and perfusion studies also found hemispheric hypoperfusion and, in some patients, perihematomal areas of decreased apparent diffusion coefficients, which were correlated with poor outcome. If these areas represented risk for secondary ischemia, BP reduction could lead to further neurological injury ^16^.

Despite this concern, early animal studies showed that controlled antihypertensive treatment in the acute period had no adverse effect on ICP or regional cerebral blood flow (CBF), as measured by radiolabeled microspheres ^17^. Additionally, human radiographic studies of the posited “penumbra” suggested that early BP control in acute ICH could be safe. Positron emission tomography (PET) was used to demonstrate that perihematomal areas of hypoperfusion were characterized by matched reduction in demand, with reduced oxygen metabolism and extraction rather than the increased oxygen extraction seen in ischemia ^18^. PET was also used to show that the use of antihypertensive medications to lower MAP in the acute period led to no further changes in global or perihematomal blood flow, suggesting preservation of autoregulation despite antihypertensive treatment ^19^. More recently, similar findings were confirmed by using computed tomography (CT) perfusion imaging in the Intracerebral Hemorrhage Acutely Decreasing Blood Pressure Trial (ICH-ADAPT) ^20^. Patients presenting with SBP of more than 150 mm Hg and moderately sized ICH within 24 hours of onset were randomly assigned to a target SBP of less than 150 mm Hg or less than 180 mm Hg. There was no difference in perihematomal CBF between groups and no association between magnitude of SBP reduction and perihematomal CBF. In addition, maximum oxygen extraction fraction (OEF[max]) and maximum cerebral metabolic rate of oxygen (CMRO2[max]) were not affected by aggressive SBP treatment ^21^. Cerebral perfusion pressure was also maintained in the perihematomal region, and the perfusion pressure did not differ by BP treatment arms or BP ^22^. Information from these studies offered to quiet the theoretical risk of major ischemia in the perihematomal area. However, substantial prospective data were not available at the time.

The American Heart Association/American Stroke Association guidelines for management of spontaneous ICH have evolved over the last few iterations, reflecting conclusions drawn from sequential studies on intensive BP treatment. Prior to publication of the 2007 guidelines ^23^, a prospective trial of 27 patients found a low rate of neurological deterioration and hematoma expansion in patients who received treatment to reduce BP below 160 systolic and 90 diastolic within 24 hours ^24^. Two clinical trials—the Intensive Blood Pressure Reduction in Acute Cerebral Hemorrhage (INTERACT) ^25^ and the Antihypertensive Treatment in Acute Cerebral Hemorrhage (ATACH) study ^26^—had just begun. In the absence of clinical trial data, guideline recommendations were hesitant, suggesting treatment for BPs over 200 mm Hg systolic or MAP over 150 mm Hg, and discretion to treat BPs over 180 mm Hg systolic or MAP over 130 mm Hg, either to a goal cerebral perfusion pressure of 60–80 mm Hg in patients with elevated ICP or to a target BP 160/90 mm Hg or MAP 110 mm Hg in those not suspected to have elevated ICP ^23^.

In the mid-2000s, a systematic review reported that elevated SBP was associated with more than fivefold odds of subsequent death or deterioration after primary ICH ^27^. A few years later, a prospective Chinese study of 1,760 patients with ICH reported a direct linear association between elevations in SBP and death and major disability ^28^. Another retrospective study, of 122 patients with ICH, found an association between BP control and neurological deterioration over the course of 24 hours ^29^. The risk of neurological deterioration—defined by comparing baseline and 24-hour Glasgow Coma Scale (GCS) scores—was significantly lower in the quartile of patients with maximum SBP drop over the course of 24 hours. These studies further highlighted the need to determine whether intervening in elevated BP could mitigate the poor outcomes seen after acute ICH, and trials ensued ( Table 1).

**Table 1.** Summary of randomized, prospective clinical trials studying acute blood pressure management in intracerebral hemorrhage.

Study (year of publication)	Patients	Number of subjects	Intervention	Primary outcome	Results
Rapid blood pressure reduction in acute ICH (2008)	Supratentorial ICH within 8 hours of symptom onset	42	MAP <110 versus MAP 110–130	Decline in NIHSS ≥2 points at 48 hours, mRS score ≤2 at 90 days, hematoma and edema expansion >30% from baseline volume on 24-hour CT	No significant differences in early neurological deterioration ( *P* = 0.55), hematoma and edema growth ( *P* = 1.0, *P* = 0.35), and clinical outcome at 90 days ( *P* = 0.43).
Intensive Blood Pressure Reduction in Acute Cerebral Hemorrhage (INTERACT) (2008)	ICH within 6 hours of symptom onset and SBP 150–220	404	SBP <140 versus SBP <180	Proportional change in hematoma volume in 24 hours, mRS score of 3–6 at 90 days	No excess neurological deterioration or other adverse events in intensively treated group, reduced rate of hematoma growth by 8% ( *P* = 0.05)
Antihypertensive Treatment in Acute Cerebral Hemorrhage (ATACH) (2010)	Supratentorial ICH within 6 hours of symptom onset and SBP ≥200	60	IV nicardipine, three tiers of SBP: 170–200 140–170 110–140	Neurological deterioration within 24 hours, serious adverse events within 72 hours	Low rate of serious adverse events and neurological deterioration among all three tiers. No difference in average SBP change between patients with and without neurological deterioration ( *P* = 0.47)
Intracerebral Hemorrhage Acutely Decreasing Blood Pressure Trial (ICH-ADAPT) (2013)	ICH within 24 hours of symptom onset and SBP ≥150	82	IV labetalol, SBP <150 versus <180	Perihematoma rCBF on CT perfusion, 2 hours after treatment	Peri-hematoma rCBF was not lower among patients randomly assigned to SBP <150 ( *P* = 0.18)
INTERACT2 (2016)	ICH within 6 hours of symptom onset, SBP 150–220	2,794	SBP <140 within 1 hours versus SBP <180	Death or mRS score >2 at 90 days	No significant change in the rate of death or major disability. Trend toward improved functional outcome on ordinal analysis. OR 0.87 (95% CI 0.75–1.01, *P* = 0.06)
ATACH-2 (2016)	Supratentorial ICH within 4.5 hours of symptom onset, SBP ≥180	1,000	SBP 110–139 versus SBP 140–179	Death or mRS score of 4–6 at 90 days	No difference in the rate of death or severe disability ( *P* = 0.72). Higher rate of renal complications in 7 days among treatment arm ( *P* = 0.002)

Blood pressure is presented in millimeters of mercury (mm Hg). CI, confidence interval; CT, computed tomography; ICH, intracerebral hemorrhage; IV, intravenous; MAP, mean arterial pressure; mRS, modified Rankin Scale; NIHSS, National Institutes of Health Stroke Scale; OR, odds ratio; rCBF, relative cerebral blood flow; SBP, systolic blood pressure.

### Early trials

In 2008, a prospective trial which randomly assigned 21 patients each to groups with target MAP of less than 110 mm Hg versus 110–130 mm Hg found no significant differences between the groups in early neurological deterioration, hematoma and edema growth, and clinical outcome at 90 days ^30^. Additionally, INTERACT was published in 2008 and provided data in the form of an open-label, randomized controlled trial of 404 patients from China, Australia, and South Korea with CT-confirmed ICH, who had SBP of 150–220 mm Hg and could be assessed and receive treatment within 6 hours of onset. Patients were randomly assigned to target BPs of either SBP of 140 mm Hg within 1 hour to continue for 7 days or SBP of 180 mm Hg in keeping with contemporaneous guidelines. Antihypertensive agents were chosen in concordance with local protocols. Results showed a trend toward attenuation of hematoma growth in the intensive treatment group, without excess neurological deterioration or other adverse events ^25^. A post-hoc analysis of the trial reported that more intensive SBP reductions in the trial were associated with less hematoma expansion ^31^. In 2010, the National Institutes of Health–funded multicenter prospective pilot clinical trial ATACH was presented. It included 60 patients who presented within 6 hours of ICH onset, in whom intravenous nicardipine was used to assess three tiers of SBP targets (170–200, 140–170, and 110–140 mm Hg) to be maintained for 24 hours. The 3-month mortality was lower than expected in all tiers, and frequencies of neurological deterioration and serious adverse events were below pre-specified safety thresholds ^26^. Overall, the pilot demonstrated feasibility and safety of early BP lowering. In a post-hoc analysis, patients who had more aggressive SBP reduction over the course of 24 hours (based on area under the curve of hourly SBP and baseline SBP) showed trends toward reduced hematoma expansion and better 3-month outcomes. ^32^. Based on this emerging available evidence, guidelines were updated to suggest that lowering SBP to 140 mm Hg in those patients presenting with SBP between 150 and 220 mm Hg was probably safe but underscored the uncertainty of efficacy until larger trials were completed ^33^.

The pilot clinical trial data were accompanied by additional retrospective and prospective observational studies. In 2012, a multicenter prospective study to evaluate the feasibility of intravenous nicardipine to treat hypertension in acute ICH was reported ^34^. On average, BPs among the 88 participants dropped from 175.4 ± 33.7 mm Hg systolic to 127.4 ± 16.7 mm Hg systolic over 6 hours of infusion. Only three (3.4%) patients had hematoma expansion, and two (2.2%) had neurological deterioration. In 2015, another study found an inverse association between relative SBP reduction (24-hour SBP compared with that on admission) and neurological deterioration (decrease in GCS or National Institutes of Health Stroke Scale scores at 24 hours compared with admission), hematoma expansion (>33% increase), and unfavorable outcome at 3 months (modified Rankin Scale (mRS) score >3) ^35^.

### Phase III clinical trials

The phase III INTERACT2 trial, which studied 2,794 patients presenting with SBP between 150 and 220 mm Hg within 6 hours of symptom onset, provided the largest body of evidence on SBP treatment to date. Those randomly assigned to the intensive treatment group had a target SBP of less than 140 mm Hg within 1 hour of randomization and for a duration of 7 days. Intravenous medications were administered per local protocols, and outcomes were assessed blinded to treatment arm. The standard treatment group had a target SBP of less than 180 mm Hg. The intensive treatment group had an odds ratio (OR) of 0.87 (95% confidence interval (CI) 0.75–1.01, *P* = 0.06) for the primary outcome of death or major disability, defined as an mRS score of more than 2 at 90 days. Although effect on the primary outcome just missed statistical significance, secondary analyses indicated that the intensive treatment group had significantly better functional recovery on an ordinal analysis of mRS scores and better physical and mental health-related quality of life on the European Quality of Life 5 Dimension (EQ-5D) scale ^36^. There were no safety concerns identified. A post-hoc analysis of INTERACT2 did find that greater reduction in SBP was associated with reduced hematoma growth (13.3 mL for less than 10 mm Hg, 5.0 mL for 10–20 mm Hg, and 3.0 mL for at least 20 mm Hg, *P* <0.001). In addition, the lowest mean hematoma growth was in patients in the intensive treatment arm who achieved a target SBP at less than 1 hour compared with those patients who achieved target SBP at later time points ^37^.

Interestingly, INTERACT2 did not show a clear relationship between outcome and time from onset to initiation of BP treatment. Of note, only one third of patients achieved the target SBP within 1 hour; half achieved the target in 6 hours. This raises the possibility that the duration of BP control, rather than the rapidity of treatment, contributed to the outcomes. There are also important populations for whom INTERACT2 did not provide data, including patients presenting with SBP of more than 220, larger hematomas, and more severe neurological deficits and patients undergoing decompressive craniectomy. Nevertheless, the trial offered strong evidence that early intensive BP lowering in the studied population did not lead to increased risk of death or serious adverse events. It demonstrated an effect of intensive treatment target on better functional recovery (although this was a secondary endpoint) as well as a trend toward decreased mortality and major disability (primary endpoint). Based on these data, the current American Heart Association guidelines were revised to state that for “ICH patients presenting with SBP between 150 and 220 mm Hg and without contraindication to acute BP treatment, acute lowering of SBP to 140 mm Hg is safe (Class I; Level of Evidence A) and can be effective for improving functional outcome (Class IIa; Level of Evidence B)” ^38^.

The most recent data reported are from the ATACH-2 trial ^39^. This randomized controlled trial enrolled 1,000 patients in the USA, Europe, and Asia who presented with primary supratentorial ICH and initial SBP of more than 180 mm Hg. Patients were initially enrolled within a 3-hour window from symptom onset, but later the window was expanded to 4.5 hours after an analysis found similar prevalence of hematoma expansion within the 3- to 4.5-hour window ^40^. Patients 18 years or older with a GCS score of 5 or more and hematoma volumes of less than 60 mL on CT scan were eligible. They were randomly assigned to either the intensive treatment group with a goal SBP of 110–139 mm Hg for 24 hours or the standard treatment group with a goal SBP of 140–179 mm Hg. Intravenous nicardipine was used according to a standard protocol: initiation at 5 mg per hour and then an increase by 2.5 mg per hour every 15 minutes as needed to a maximum of 15 mg per hour. After this, if the SBP was still above target, then intravenous labetalol was used (and if it was not available, intravenous diltiazem or urapidil was used).

The primary outcome in ATACH-2 was death or moderately severe to severe disability (mRS score >3) at 3 months. The trial was terminated for futility before reaching the proposed enrollment target of 1,280 subjects, which was calculated to identify a 10% difference between groups. Analysis of the first 1,000 subjects recruited showed that the absolute difference in the rate of death and disability was similar between the two treatment groups (38.7% in the intensive treatment group and 37.7% in the standard treatment group; OR 1.04, CI 0.85–1.27). There was also no difference between groups on the ordinal analysis of the mRS score. The rate of hematoma expansion was slightly lower in the intensive treatment group (19% versus 24%, not significant); however, quality-of-life assessment using EQ-5D also did not demonstrate significant difference between the two groups. There was no difference in the rate of serious adverse events within 72 hours between the two groups; however, the rate of non-serious renal adverse events within 7 days was higher in the intensive treatment group (9% versus 4%). In addition, there were higher rates of serious adverse events within 3 months in the intensive treatment group (25.6% versus 20.0% in the standard treatment group; adjusted relative risk 1.30, CI 1.00–1.69, *P* = 0.05).

Not surprisingly, there was a higher proportion of patients with treatment failure in the intensive treatment group than the standard treatment group. Primary treatment failure, defined as not reaching the target SBP within 2 hours after randomization, occurred in 12.2% of patients in the intensive treatment group versus 0.8% of the standard treatment group. Secondary treatment failure was defined as the hourly minimum SBP greater than the target upper limit for two consecutive hours during the period of 2 to 24 hours after randomization. This occurred in 15.6% of patients in the intensive treatment group versus 1.4% in the standard treatment group. The rate of treatment failure leaves open the possibility that a greater outcome effect could have been achieved if more patients had met the treatment goal, although potentially also at the cost of greater rates of adverse events.

Recent meta-analyses ^41, 42^ of the major clinical trials—including INTERACT 1 and 2, the feasibility and safety study by Koch *et al*. ^30^, ICH-ADAPT, and ATACH-2—indicate that intensive BP-lowering treatment is associated with a modest and non-significant trend toward lower 3-month mortality and dependency as well as reduced hematoma expansion. Though non-significant, the observed trend is intriguing. Further study is needed to better understand the biology underlying the relationships between BP reduction, hematoma expansion, and outcome after ICH, which in turn may inform optimal patient selection for intensive BP reduction.

## Remaining questions

### Timing, target, and duration of control

The two largest clinical trials—INTERACT2 and ATACH-2—used SBP of less than 180 mm Hg as the standard treatment target, yet the intensity in the intervention arm and the time to achievement of SBP goals differed between the trials. In ATACH-2, the mean minimum SBP values during the first two hours were 128.9 ± 16 mm Hg in the intensive treatment group and 141.1 ± 14.8 mm Hg in the standard treatment group, and 88% of patients met the SBP goal within 2 hours ^39^. Even in the standard treatment group, BPs remained under 160 mm Hg for the first 24 hours. Interestingly, in INTERACT2, the mean SBP of the intensive treatment group was 150 mm Hg within the first hour (compared with 164 mm Hg in the standard treatment group), and only 30% of patients in the intensive arm of the trial achieved the target SBP level of less than 140 mm Hg within 1 hour ^36^. Thus, the INTERACT2 trial in effect tested a more moderate approach to control of hypertension than ATACH-2 and showed more promising results on clinical outcomes. Neither trial was designed to inform whether SBP of 140–160 mm Hg would have different outcomes from SBP of 160–180 mm Hg, assuming that target BPs can be achieved and maintained. Subgroup analyses to address outcome differences between groups as defined by their achieved BPs within the range of SBP of 140–180 mm Hg could be informative. In addition, SBP goals were maintained for 7 days in INTERACT2 but for only 24 hours in ATACH-2, raising important questions about optimal timing and duration of BP control and the effects of these variables on patient outcomes.

With consideration to the timing of initiation of BP treatment, pre-hospital screening, assessment, and initiation of treatment are of great interest. Two trials have demonstrated the feasibility of executing an ambulance-based, paramedic-initiated randomized controlled trial. In Paramedic Initiated Lisinopril For Acute Stroke Treatment (PIL-FAST) ^43^, 14 patients with suspected acute stroke and hypertension were recruited and randomly assigned to a first dose of lisinopril versus placebo; four patients completed 7 days of study treatment. In the Rapid Intervention with Glyceryl Trinitrate in Hypertensive Stroke Trial (RIGHT) ^44^, 41 of 80 screened patients were enrolled and successfully randomly assigned to transdermal glyceryl trinitrate versus placebo. SBP between the groups was significantly different at 2 hours ( *P* = 0.03), and there was no difference in rates of serious adverse events or death. Pre-hospital identification of definitive ICH, if achievable, could allow further study on the effects of pre-hospital initiation of antihypertensive treatment specifically in this population.

The challenges that exist in applying the available data to individual clinical situations are universal. For BP management in ICH, conclusions from the trials must be extrapolated when patients do not fall within the description of the study patient populations. Patients comprising the majority of subjects in the aforementioned studies had small to moderate-sized hematomas, leaving those with large and more severe hemorrhages an understudied population. Over half of participants in both INTERACT2 and ATACH-2 were recruited from Asian countries. However, there were no differences found in treatment effect among Asian versus non-Asian subjects in either study. In terms of intervention, BP parameters and choice of intervention varied among the studies and depended on the resources available to practicing clinicians; thus, conclusions from the different trials may or may not be generalizable.

### Patient selection issues

The decision to pursue more aggressive treatment may be informed by methods useful for identifying patients most likely to benefit. Identifying those who may be at particular risk of hematoma expansion could sway the approach to treating hypertension. An interesting body of research has focused on the “spot sign”, or the appearance of contrast extravasation on CT angiography, which is thought to represent an area of active bleeding. This effect has been found to be independently associated with hematoma expansion ^45, 46^ and mortality ^47^. Other studies have highlighted additional radiographic predictors of hematoma growth, including spot sign number ^48^; the presence of fluid levels, density heterogeneity, and margin irregularity ^49^; the black hole sign ^50^; and the blend sign ^51^. If radiographic predictive features for hematoma expansion prove robust, they may offer a selection tool for early intensive BP treatment. One such study (SCORE-IT) is testing this hypothesis by using the spot sign in the ATACH-2 study ^52^.

Alternatively, neuroimaging may identify patients at risk of cerebral ischemic events with aggressive BP control. There is a growing literature on the occurrence of diffusion-weighted imaging (DWI) lesions, remote from the primary site of injury and delayed from hemorrhage onset, on the MRIs of an estimated 25% of patients with ICH. Mechanistic theories suggest a “stroke-prone” state after hemorrhagic or ischemic stroke, composed of an enhanced thrombotic milieu or impaired hemodynamic regulation (or both) amidst pre-existing vascular pathology, making distant brain regions potentially susceptible to secondary injury in the setting of rapid BP lowering ^53^. Such DWI lesions have been found to be more prevalent in patients with acute SBP lowering ^53, 54^ and patients with cerebral amyloid angiopathy ^55, 56^ and have been associated with poor functional outcomes ^57^. In an effort to prospectively study the relationship between BP management and DWI lesions after ICH, the ongoing randomized trial ICH ADAPT II will study patients randomly assigned to an SBP target of less than 140 versus less than 180, with MRI performed on days 2, 7, and 30 to assess the impact of hypertensive therapy on DWI lesions, both at 48 hours and cumulatively over 30 and 90 days. Secondary outcomes will include absolute hematoma growth and prediction of DWI lesion incidence as well as mortality, functional outcome, and cognitive status ^58^. Factors besides acute BP lowering may be associated with DWI lesions, however. A study of 600 patients with primary ICH found DWI lesions in 26.5% of cases, and associated variables after controlling for race/ethnicity, leukocyte count, and acute in-hospital antihypertensive treatment included higher first recorded SBP as well as greater change in MAP, microbleeds, white matter hyperintensity score, and lower age ^59^. The association of such imaging markers with different pathologies underlying hemorrhage (that is, cerebral amyloid angiopathy versus hypertensive spontaneous ICH) raises an important distinction for patient selection that has been unaddressed thus far. If the risks and benefits of aggressive BP control vary depending on the underlying pathology, that patients with decidedly different etiologies for spontaneous ICH have been studied as one population may account for some of the difficulties finding evidence of therapeutic benefit. Systematic studies using neuroimaging may assist in delineating optimal selection strategies for SBP target in individual patients.

### Medication selection

Data are even sparser on specific medication selection; one study has compared nitroprusside and nicardipine and found lower rates of mortality among those who received nicardipine ^60^. However, a small study of nine patients who received nicardipine and were monitored with transcranial Doppler found evidence of paradoxical intracranial vasoconstriction, as characterized by prominent systolic peak and dicrotic notch, pronounced systolic deceleration, and elevated pulsatility index during nicardipine infusion, thus contradicting the expected autoregulatory response to decreasing BP ^61^. Without a large body of evidence for one agent over another, the most recent guidelines suggest that an agent be chosen on the basis of practicability, pharmacological profile, potential side effects, and cost ^38^. Relevant to this question are animal studies that have shown that inflammation contributes to secondary injury in ICH, making the various factors involved in promoting inflammatory pathways attractive potential treatment targets ^62^. There are data to suggest that activation of the sympathetic nervous system leads to increased inflammation ^63^. Motivated by this concept, an analysis of the placebo-arm patients in a randomized trial testing a potential neuroprotective agent found that the use of anti-adrenergic medications (beta-blockers and alpha[2]-agonists) was shown to be associated with less edema on 72-hour imaging after hemorrhage volume and BP were controlled for ^63^. In a retrospective study investigating the association of beta-blocker use with mortality among patients in a prospectively collected ICH database, in-patient beta-blocker use was independently associated with reduced rates of in-hospital and 3-month mortality. However, univariate and multivariate analyses comparing outcomes among patients who received beta-blocker therapy versus other antihypertensive classes of medications failed to show any class-specific difference ^64^. Potential benefits of different classes of medications on other mechanisms of injury have not been extensively studied. Differences in medication classes used in INTERACT2—primarily alpha(1)-antagonist (urapidil), calcium channel antagonists (nicardipine and nimodipine), and mixed alpha- and beta-antagonists (labetalol) and ATACH-2 (nicardipine)—may also have contributed to outcomes, although this remains speculative.

### Blood pressure variability

Control of BP variability did emerge from a secondary analysis of INTERACT2 as a priority in management. When serial BP measurements in two phases of treatment (first 24 hours and days 2–7) were analyzed for variability defined as the standard deviation of SBP, categorized into quintiles, a significant linear association was found between variability and the primary outcome of death or major disability at 90 days (OR 1.41, 95% CI 1.05–1.90; *P* = 0.0167 for the first 24 hours and OR 1.57, 95% CI 1.14–2.17; *P* = 0.0124 for days 2–7). Similar associations were seen for the secondary outcome of an ordinal shift in mRS score at 90 days ^65^. The findings were also reported in a retrospective cohort study ^66^. Another cohort study measured parameters, including diastolic BPs, pulse pressures, and MAPs, and found that higher mean pulse pressures were significantly associated with in-hospital mortality ^67^. Secondary analyses addressing this same topic in the ATACH-2 population will be of great interest. It may emerge that avoidance of SBP spikes and acute BP troughs is as important as the specific target chosen if not more so. Furthermore, the use of SBP versus MAP in the different trials raises uncertainty as to which would be the better physiologic marker of the biological effects of BP variability in ICH. Further study to characterize not only the optimal measurement target but also the biological correlates of changes in MAP versus SBP could be important to understanding the physiologic mechanisms underlying outcome effects of different approaches to intensive BP management.

## Conclusions

Research on BP management in acute ICH over years of study has focused on the mechanics of hematoma expansion, perihematomal physiology, mechanisms of secondary injury, and the relationship between BP targets and patient outcomes. Recent trials have offered the needed data on the safety and potential efficacy of moderately aggressive SBP control in acute ICH. However, the precise interactions between patient selection and the timing and duration of different interventions and outcomes remain intriguing and require further definition.

## Abbreviations

ATACH, Antihypertensive Treatment in Acute Cerebral Hemorrhage; BP, blood pressure; CBF, cerebral blood flow; CI, confidence interval; CT, computed tomography; DWI, diffusion-weighted imaging; EQ-5D, European Quality of Life 5 Dimension; GCS, Glasgow Coma Scale; ICH, intracerebral hemorrhage; ICP, intracranial pressure; INTERACT, Intensive Blood Pressure Reduction in Acute Cerebral Hemorrhage; MAP, mean arterial pressure; MRI, magnetic resonance imaging; mRS, modified Rankin Scale; OR, odds ratio; PET, positron emission tomography; SBP, systolic blood pressure.
